# Non-invasive assessment of cerebral hemoglobin parameters in intracranial dural arteriovenous fistula using functional near-infrared spectroscopy—A feasibility study

**DOI:** 10.3389/fnins.2022.932995

**Published:** 2022-11-14

**Authors:** Santhakumar Senthilvelan, Santhosh Kumar Kannath, Karumattu Manattu Arun, Ramshekhar Menon, Chandrasekharan Kesavadas

**Affiliations:** ^1^Neuroradiology Division, Department of Imaging Sciences and Interventional Radiology, Sree Chitra Tirunal Institute for Medical Sciences and Technology, Thiruvananthapuram, India; ^2^Department of Neurology, Sree Chitra Tirunal Institute for Medical Sciences and Technology, Thiruvananthapuram, India

**Keywords:** cerebral hemodynamics, dural arteriovenous fistula (DAVF), functional imaging, functional near-infrared spectroscopy (fNIRS), intracranial venous hypertension

## Abstract

**Purpose:**

The purpose of this study was to assess the feasibility of non-invasive assessment of cerebral hemodynamics using functional near-infrared spectroscopy (fNIRS) in patients with intracranial dural arteriovenous fistula (DAVF) and to correlate the hemodynamic changes with definitive endovascular treatment.

**Methodology:**

Twenty-seven DAVF patients and 23 healthy controls underwent 20-mins task-based functional near-infrared spectroscopy and neuropsychology evaluation. The mean change in the hemoglobin concentrations obtained from the prefrontal cortex was assessed for oxyhemoglobin, deoxyhemoglobin, and oxygen saturation (HbO, HbR, and SO_2_, respectively). The fNIRS data were analyzed and correlated with improvement in neuropsychology scores at 1-month follow-up.

**Results:**

There was a significant reduction in HbO in the patient group, while it increased in controls (−2.57E−05 vs. 1.09E−04 mM, *p* < 0.001). The reduced HbO significantly improved after embolization (−2.1E−04 vs. 9.9E−04, *p* = 0.05, *q* = 0.05). In patients with aggressive DAVF (Cognard 2B and above), the change was highly significant (*p* < 0.001; *q* = 0.001). A moderate correlation was observed between MMSE scores and HbO changes (ρ = 0.4).

**Conclusion:**

fNIRS is a useful non-invasive modality for the assessment of DAVF, and could potentially assist in bedside monitoring of treatment response.

## Introduction

Dural arteriovenous fistula (DAVF) represents ~10–15% of all intracranial arteriovenous shunting pathologies (Tanaka, 2019). The two most disabling complications of DAVF are intracerebral hemorrhage (ICH) and non-hemorrhagic neurological deficit (NHND). The incidence of such complications tends to increase with the grade of DAVF and ranges from < 2% in non-aggressive DAVF to as high as 8.1 and 6.9% for ICH and NHND, respectively, in aggressive DAVF (Borden et al., 1995; Cognard et al., 1995; Davies et al., 1997; Satomi et al., 2002; van Dijk et al., 2002; Gross and Du, 2012; Shah et al., 2012).

NHND includes symptoms related to venous congestion, such as cognitive impairment and dementia, seizures, or dysphasia, which can range from subtle to severe. While patients with benign symptoms such as tinnitus can be managed conservatively, those with NHND or ICH require definitive treatment. ICH can be readily identified using conventional imaging modalities, but subtle NHNDs are difficult to detect *via* clinical examination or imaging. Neuropsychological examination, although not routinely performed during the evaluation of DAVF, may be an effective method for uncovering subtle cognitive deficits (Racine et al., 2008). Early identification of NIHD can prevent progression to severe clinical deterioration, especially in benign DAVFs, which are usually managed conservatively. Thus, a non-invasive modality to identify subtle NHNDs related to venous congestion can assist in early and effective treatment, and the same modality could be also utilized for follow-up assessment.

Functional near-infrared spectroscopy (fNIRS) is an optical imaging technique that utilizes infrared rays emitted at various wavelengths. The reflected light is used to measure chromophores, such as hemoglobin and cytochrome oxidase. Several types of fNIRS are currently in use, including continuous wave fNIRS (cw-fNIRS), frequency domain spectroscopy, and time domain spectroscopy. cw-fNIRS, the most common of the types listed above, is based on the principle of the emission of infrared light at two specific wavelengths, its absorption by the chromophores (oxyhemoglobin and deoxyhemoglobin), and its reflection. The reflected light is then measured, which gives the absorption coefficients of oxyhemoglobin and deoxyhemoglobin. The change in absorption between the task phase and the rest phase can then be estimated from these measured coefficients. This change, in turn, describes the change in the concentrations of oxyhemoglobin and deoxyhemoglobin. fNIRS has already been shown to have a role in the diagnosis and management of various neurological disorders, such as Alzheimer's dementia, epilepsy, neurotrauma, and stroke rehabilitation (Hock et al., 1996; Watanabe et al., 2002; Plenger et al., 2016; Arun et al., 2020).

Because cognitive impairment is thought to be linked to venous hypertension secondary to DAVF, we hypothesized that fNIRS could aid in estimating cerebral hemodynamics at the bedside and correlating the observable changes after effective treatment of fistula. In this study, we used task-based fNIRS in all patients with DAVF who were evaluated with a standard battery of neuropsychological tests. Subsequently, we investigated the correlation between the hemodynamic variations and cognitive status at presentation and the endovascular management of DAVF.

## Materials and methods

### Patient population

**Figure 1:**
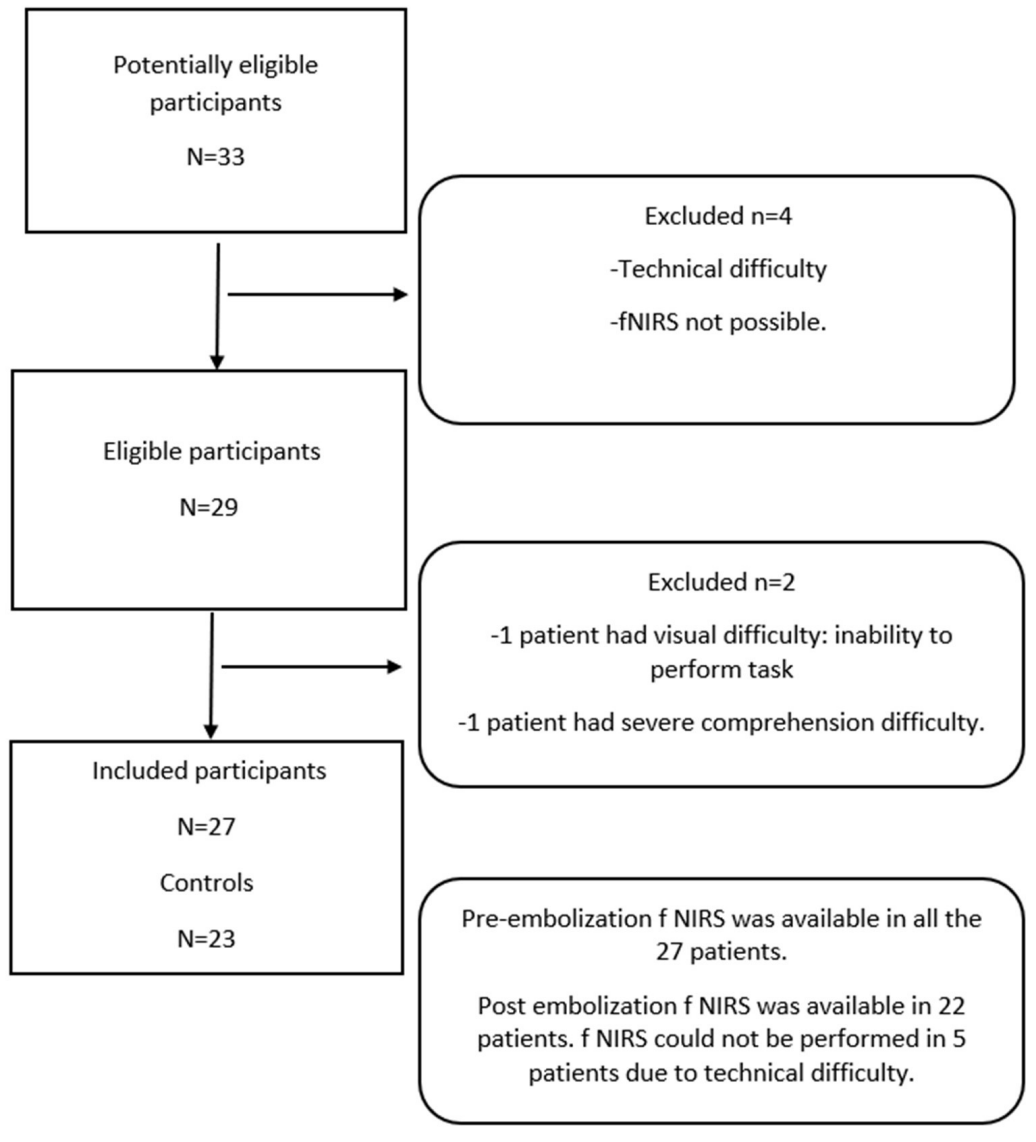
Patient flow diagram: A total of 27 patients and 23 age-matched controls were included in the study.

Between October 2018 and September 2020, 27 consecutive patients with intracranial DAVF proven *via* imaging studies and 23 healthy controls who met the inclusion criteria were prospectively enrolled in the study. Patients were included if they were older than 18 years of age and were able to comprehend simple commands. Patients who were not able to understand simple commands and who had coexisting neurological abnormalities (as inferred from the available clinical and imaging data) were excluded from the study. The participant selection is presented in Figure 1.

Healthy individuals without a known prior history of neurological or systemic disorders were included as controls. Informed consent was obtained from all study participants. All procedures were performed in compliance with the Declaration of Helsinki, and institutional ethics committee approval was obtained before commencing the study (Institutional Ethics Committee, Sree Chitra Tirunal Institute for Medical Sciences and Technology, IEC number: 1241).

### fNIRS protocol

#### Experimental design

The study was conducted in three parts. In the first part, participants were instructed to gaze at a fixation point on the computer screen without performing any task. Subsequently, a predesigned Stroop task was done twice (two trials), with intervening periods of activity and rest. The durations of activity and rest were equal, each lasting 20 s.

#### Stroop task

The Stroop task was used because it assesses cognitive functions, is easy to perform, is reproducible, and is objective in nature. The Stroop task involves showing the participant words depicted in various colors and asking the participant to identify only the color of the word, not the word itself.

#### Paradigm design

**Figure 2:**
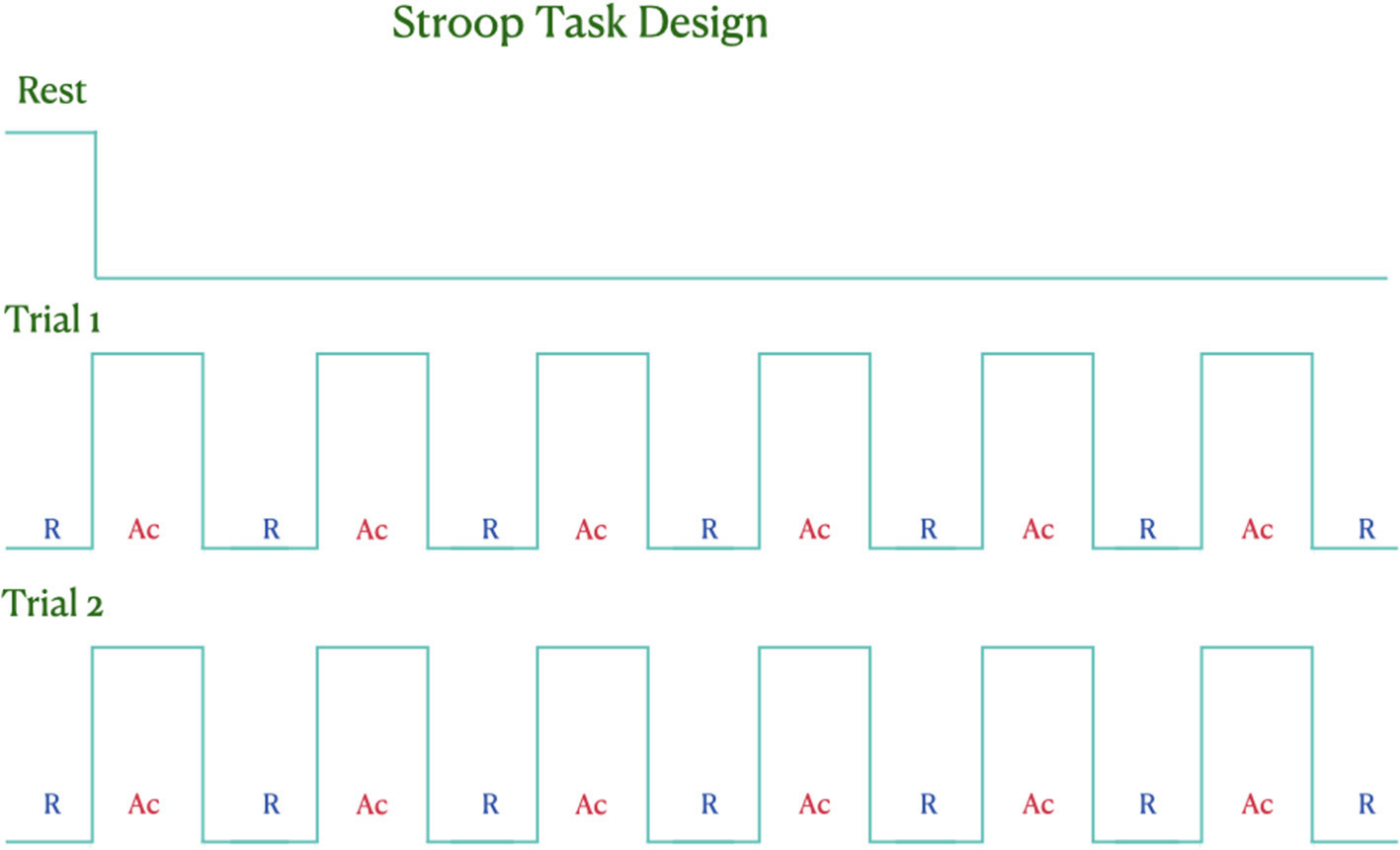
Schematic diagram of the paradigm used in our study. The participants underwent an initial resting phase of 3 min, followed by two trials of activity with intermittent rest periods.

A synchronized paradigm was required for accurate analysis. The paradigm was predesigned using NIRSTIM 4.0 software provided by NIRX LLC (NIRX Medical Technologies, Berlin, Germany). NIRSTIM was chosen as it allows the paradigm to be launched directly from the acquisition software, thus ensuring synchronization with the acquired data. The paradigm is represented in Figure 2.

### Data acquisition

**Figure 3:**
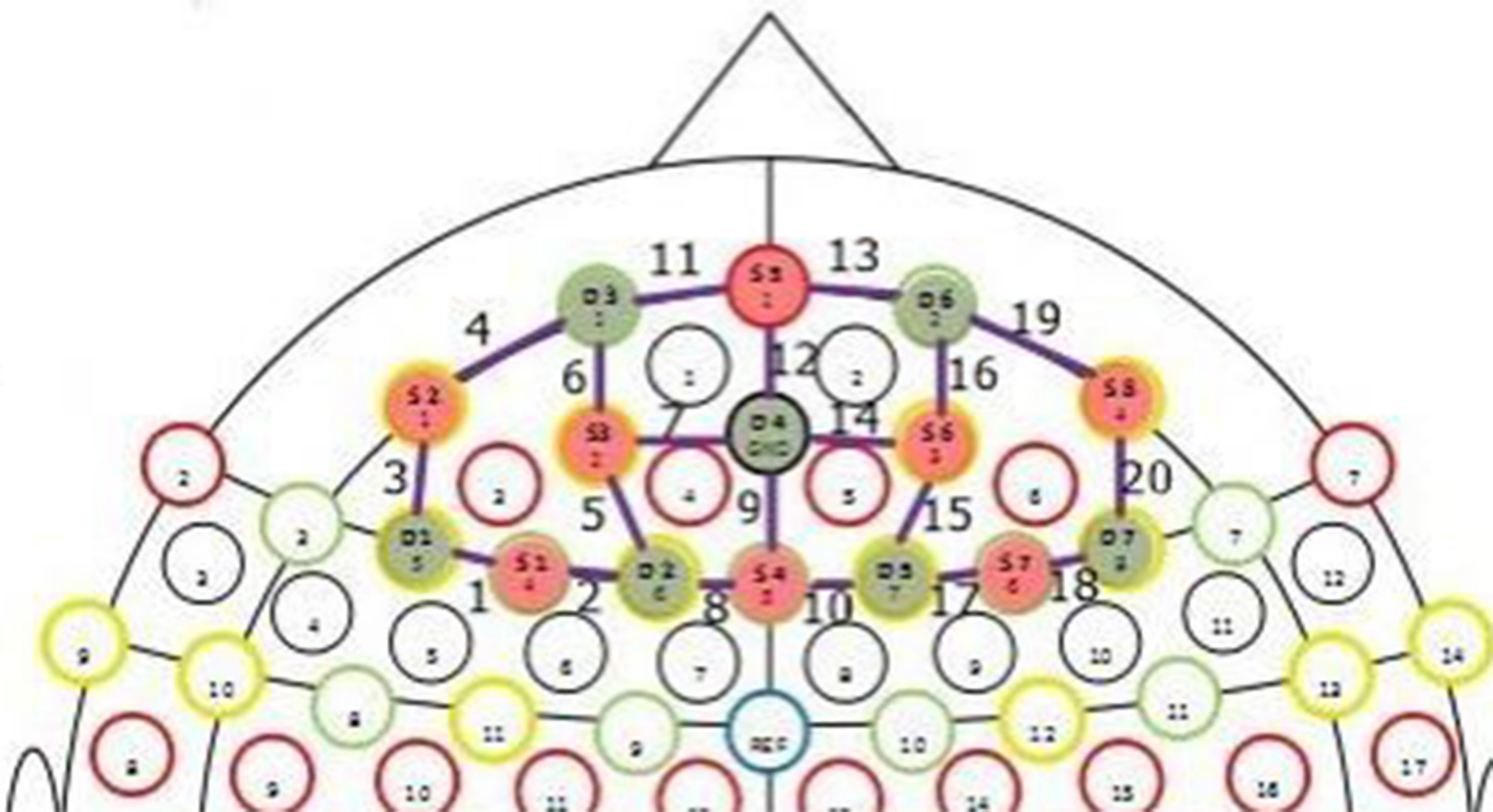
Photograph of the prefrontal 8 × 8 montage used in the study. Channels are numbered from 1 to 20 in the image. Channels correspond to parts of the prefrontal cortex.

The fNIRS data were acquired using the NIRSport system (NIRx Medical Technologies LLC, Berlin, Germany), which has eight sources and eight detectors, at a sampling rate of 7.825 Hz. The two wavelengths used were 760 and 850 nm. The prefrontal cortices of both hemispheres were covered using eight sources and seven detectors, thus forming a total of 20 channels (Figure 3). Each study participant was comfortably seated in a chair. The optodes were placed in on the participant's head such that they were in direct contact with the scalp. Any intervening hair was gently displaced, and the optodes were held together with a fabric NIRS cap specifically designed for this examination. Direct skin–optode contact was ensured for good signal quality.

The data were acquired using in-built software and analyzed using the MATLAB-based NIRSLab software (NIRx Medical Technologies, LLC, Berlin, Germany). The data were preprocessed after checking the quality of the signal obtained. Signal discontinuities and sudden spikes were removed. The band-pass filter was then placed at 0.01–0.2 Hz. After preprocessing the data, the hemodynamic states were computed. The mean block averaged data were calculated and used for the statistical analyses. Differential path length factors (DPFs) were obtained from previous studies to account for the scattered radiation (Kohl et al., 1998). The data analysis was based on the modified Beer–Lambert law and took the scatter factor into account.

### Neuropsychological tests

The patients underwent the Mini-Mental Status Examination (MMSE) and the Addenbrooke's Cognitive Examination III (ACE-III) before and 1 month after the embolization. The controls also underwent these tests. The tests were performed by a trained clinical neuropsychologist with 5 years of experience in conducting and interpreting neuropsychological examinations.

### Embolization procedure

After a diagnosis of intracranial DAVF using digital subtraction angiograms, a definitive embolization procedure was planned within 4 weeks of the diagnosis if higher-grade DAVF or significant clinical symptoms were observed. The embolization was performed transarterially using liquid embolic agents, such as Onyx^TM^ (Medtronic, Minneapolis, USA) or Squid^TM^ (Balt, Montmorency, France). Post-procedure, the patient was started on intravenous heparin and subsequently low-molecular-weight heparin or warfarin, if significant venous stasis was observed after the embolization.

The embolization outcome was classified as follows: (1) complete, if there was complete resolution of DAVF; (2) significant, if there was a complete cessation of cortical venous reflux but the downgraded (benign) residual fistula persisted; (3) partial, if partial reduction in the fistula was achieved, with the presence of cortical venous reflux. These outcomes were assessed independently by an observer not involved in the fNIRS data collection. Circulation time was recorded before and after the procedure.

Magnetic resonance imaging (MRI), fNIRS, and neuropsychological examination were each performed once before embolization (fNIRS was done precisely after the initial diagnostic angiogram in which DAVF was proven). Postembolization, the patient underwent repeat MRI, fNIRS, and neuropsychological examination after 1 month. The total time required to acquire and process a patient's fNIRS data was ~1 h.

### Data collection

Data on the changes in oxyhemoglobin, deoxyhemoglobin, and total hemoglobin were collected. The change in hemoglobin levels indicates the change in chromophore concentration after an activity (namely, the Stroop task) with respect to the resting phase. The absolute values of the chromophore concentration could not be measured using cw-fNIRS. The change in hemoglobin values was measured in millimoles (mM). This change is extremely small, usually on the order of E−05 or E−04 (i.e., 10^−5^ or 10^−4^) millimoles. Hence, for example, a value of about −0.00021 is represented as −2.1E−04. These changes were measured in patients at rest and after the task, before and after embolization. Similarly, changes in chromophore concentration were measured at rest and after the task in the control group. Changes in the regional oxygen saturation were also measured as a percentage.

The neuropsychology scores included were the MMSE and ACE III scores. The MMSE examination results were scored from 0 to 30, and the ACE III examination results were scored from 0 to 100.

### Statistical analyses

Preprocedural and postprocedural fNIRS data were analyzed using a paired *t*-test. Patients and controls were assessed using an unpaired *t*-test, which was also used to analyze the fNIRS data and the angiographic parameters. The normality of the data was assessed using Shapiro–Wilk and Kolmogorov–Smirnov tests. The fNIRS data followed normal distribution, whereas the neuropsychology MMSE scores did not follow normal distribution. The pre-embolization neuropsychology data were evaluated using the Mann–Whitney U-test. Pre-embolization and postembolization neuropsychology data were assessed using the Wilcoxon signed-rank test. The neuropsychology scores and fNIRS data were evaluated using the Spearman rho correlation test. Dichotomous data were analyzed using the chi-square test and Fisher's exact test. A two-sided *p* < 0.05 was considered significant for each test. The corrected *p*-value (*q*-value) was obtained using the false discovery rate (FDR) as we used multiple pairwise comparison analysis. All statistical analyses were performed using SPSS software, version 25.0.

## Results

A total of 27 patients with intracranial DAVF and 23 healthy volunteers were recruited. The mean age of the patient cohort was 48 years, and a male preponderance was noted (23:4) (Table 1). The most common clinical symptom of DAVF was headache (70%), and cognitive impairment was seen in 11 patients (40.7%). The patients' median MMSE score at the time of diagnosis was 25 (range 9–29), whereas the controls did not display abnormal MMSE scores (median 30, range 24–30). The median ACE III score in the patient group was 76 (range 25–86), whereas the median score in the control group was 94 (range 91–100).

**Table 1:** Demographic features of the study participants.

**Demographic**	**Patients**	**Controls**	***p***
Age in years (mean ± SD)	48 ± 13 (24–70)	45 ± 17.8 (20–73)	0.818
Gender (percent)	23 males (85.2%); 4 females (14.8%)	16 males (69.6%); 7 females (30.4%)	0.557
Risk factors^#^ (DM, HTN, CVT, trauma, hypercoagulable states)	19/27 (70%)	9/23 (40%)	0.192
MMSE scores^#^ (Median ± range)	25 ± 20	30 ± 1	**< 0.001 (*****q*** **<** **0.001)**
ACE III scores^#^ (Median ± range)	76 ± 61	94 ± 9	**< 0.001(*****q*** **<** **0.001)**

# DM, diabetes mellitus; HTN, hypertension; CVT, cerebral venous thrombosis; MMSE, Mini-Mental Status Examination; ACE III, Addenbrooke's Cognitive Examination III.

Bold represents statistical significance.

MRI was performed in all patients, and digital subtraction angiography (DSA) was performed in 26 patients (96.3%). One patient passed away before the angiographic study because of hemorrhage. Embolization of DAVF was performed in 25 patients (92.6%) (Table 1).

Baseline (pre-embolization) fNIRS data were available for all patients, and postembolization data were available for 22 patients.

### Before the treatment

**Figure 4:**
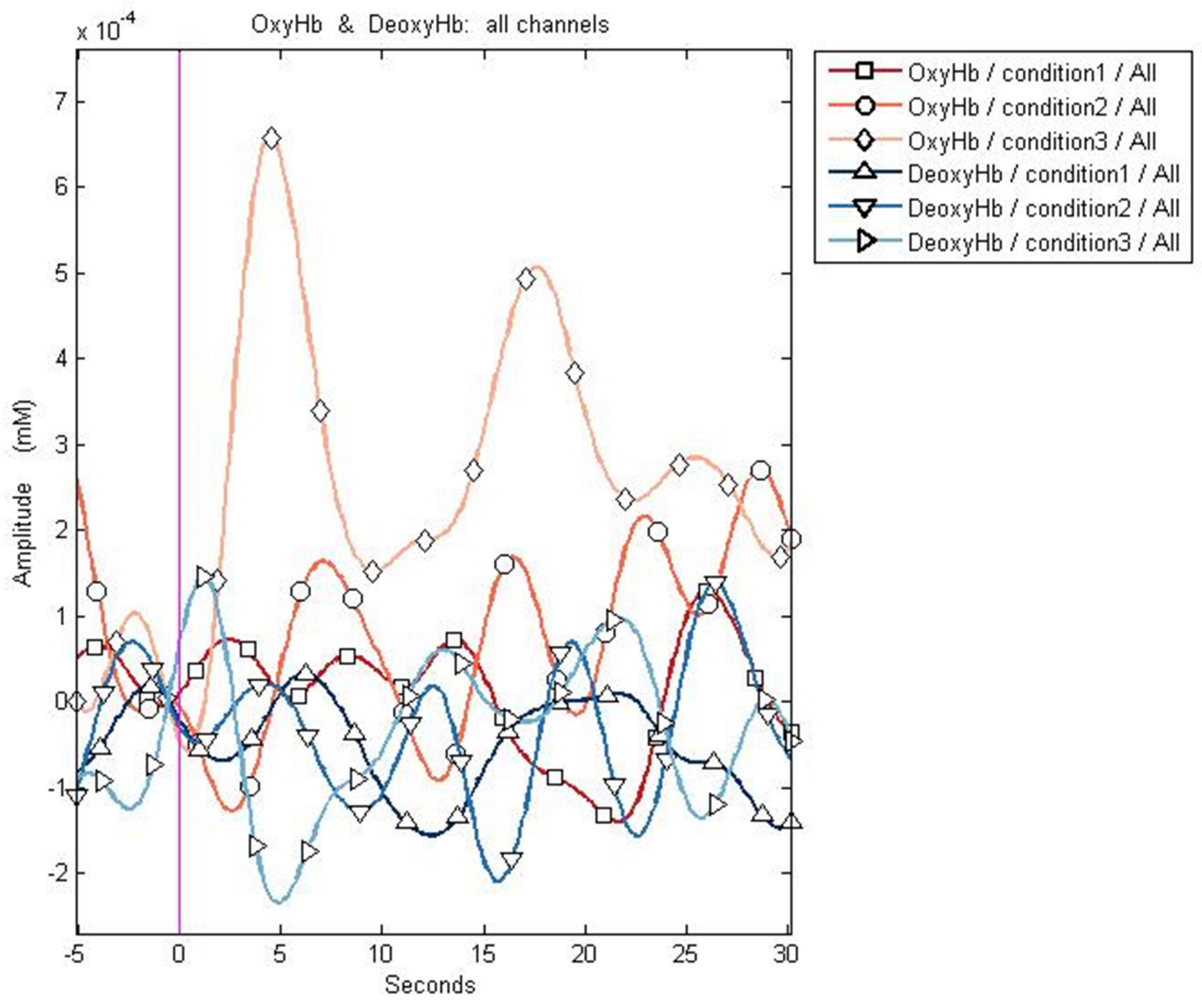
Graphical representation of the changes in oxy- and deoxyhemoglobin in a control. The control shows an increase in oxyhemoglobin (represented by diamonds and circles) after a task, while no significant change in deoxyhemoglobin is observed. Condition 1: resting phase; Condition 2: activity (trial 1); Condition 3: activity (trial 2).

As expected, the control group demonstrated a consistent increase in oxyhemoglobin (mean: 1.1E−04 ± 1.0E−04 mM), reduction in deoxyhemoglobin (mean: −4.81E−05 ± 6.84E−05 mM), and increase in regional oxygen saturation (mean: 3.58E−02% ± 1.20E−01%). However, the patient cohort showed a consistent reduction in oxyhemoglobin (mean: −2.6E−05 ± 2.1E−04 mM), reduction in deoxyhemoglobin (mean: −3.48E−05 ± 4.66E−05 mM), and increase in regional oxygen saturation (mean: 2.32E−02 ± 1.04E−01 mM). The changes in oxyhemoglobin between the patients and controls were significant, whereas the changes in deoxyhemoglobin and regional oxygen saturation were not found to be significant (the *p*-values for oxyhemoglobin, deoxyhemoglobin, and regional oxygen saturation were 0.009, 0.42, and 0.69, respectively; Table 2). The graphical representation of the hemoglobin parameters for the control group is presented in Figure 4.

**Table 2:** Task-based fNIRS changes in cerebral hemodynamics between patients and controls.

	**Task-based change in**		**Task-based change in**		**Task-based change in regional**	
	**oxyhemoglobin (mM** ^*^ **)**		**deoxyhemoglobin (mM)**		**oxygen saturation (%)**	
	**Mean ±2 SD**	***p***	**Mean ±2 SD**	***p***	**Mean ±2 SD**	***p***
Patients (*n* = 27)	−2.6E−05 ± 2.1E−04	**0.009 (** ***q*** **: 0.01)**	−3.48E−05 ± 4.66E−05	0.42	2.32E−02 ± 1.04E−01	0.69
Controls (*n* = 23)	1.1E−04 ± 1.0E−04		−4.81E−05 ± 6.84E−05		3.58E−02 ± 1.20E−01	

* mM, millimoles. Bold represents statistical significance.

While analyzing the DAVF subgroups, it was noted that the reduction in oxyhemoglobin was found to be more pronounced in patients with aggressive fistula (Cognard grade ≥ 2B) (mean: −6.9E−05 ± 1.4E−04 mM vs. −2.6E−05 ± 2.1E−04, *p* = 0.001, *q* = 0.001) and in those with cognitive symptoms (mean: −5.4E−05 ± 1.2E−04 mM vs. −2.6E−05 ± 2.1E−04, *p* = 0.001, *q* = 0.001) (Table 3).

**Table 3:** Task-based fNIRS changes in cerebral hemodynamics among patients before and after the embolization procedure.

**Patient participants**	**Pre-embolization**			**Postembolization**			***p***		
	**Oxy-Hb**	**Deoxy-hb**	**Regional saturation**	**Oxy-Hb**	**Deoxy-hb**	**Regional saturation**	**Oxy-Hb**	**Deoxy-hb**	**Regional saturation**
All patients (*n* = 22) (20 aggressive and 2 non-aggressive)	−2.1E−04 ± 2.3E−04	−3.1E−05 ± 5E−05	1E−02 ± 1.1E−01	9.9E−04 ± 1.2E−04	−8E−06 ± 5.6E−05	4.2E−02 ± 7E−02	**0.05 (** ***q*** **: 0.05)**	0.21	0.24
Aggressive fistula (Cognard ≥ 2B) (*n* = 20)	−6.9E−05 ± 1.4E−04	−2.7E−05 ± 5E−05	1.2E−02 ± 1.1E−01	1.1E−04 ± 1.3E−04	−8.7E−06 ± 5.8E−05	4.3E−02 ± 7.4E−02	**0.001 (** ***q*** **: 0.001)**	0.40	0.30
Cognitive complaints (*n* = 15)	−5.4E−05 ± 1.2E−04	**–**1.3E−05 ± 4.4E−05	1E−02 ± 1.1E−01	1.1E−04 ± 1E−04	−4E−06 ± 5.2E−05	4.2E−02 ± 7E−02	**0.001 (** ***q*** **: 0.001)**	0.18	0.8

Bold represents statistical significance.

The MMSE and ACE III scores were correlated with the changes in the oxyhemoglobin data. The reduction in oxyhemoglobin showed a weak positive correlation with the reduction in the MMSE score in the patient group (Spearman's correlation coefficient = 0.4, *p* = 0.04), whereas the ACE III scores were not significantly correlated with the pre-embolization changes in oxyhemoglobin values.

### After the treatment

The patient cohort showed a consistent increase in oxyhemoglobin after the embolization (mean: 9.9E−04 ± 1.2E−04 Mm, *p* = 0.05). However, the reduction in deoxyhemoglobin (mean: −8E−06 ± 5.6E−05 mM) and increase in regional oxygen saturation (mean: 4.2E−02% ± 7E−02%) after embolization were not found to be significant (Table 3). In the subgroup analysis, the patients with aggressive fistula and cognitive complaints demonstrated an extremely significant increase in oxyhemoglobin (mean value in the aggressive fistula subgroup: 1.1E−04 ± 1.3E−04 mM; mean value in the cognitive complaints subgroup: 1.1E−04 ± 1E−04 mM, *p* = 0.001). However, the changes in deoxyhemoglobin and regional oxygen saturation between these subgroups were not significant (the *p*-values of deoxyhemoglobin and regional oxygen saturation for patients with aggressive fistula were 0.40 and 0.30, respectively, whereas those for patients with cognitive symptoms were 0.18 and 0.8, respectively) (Table 3).

**Figure 5:**
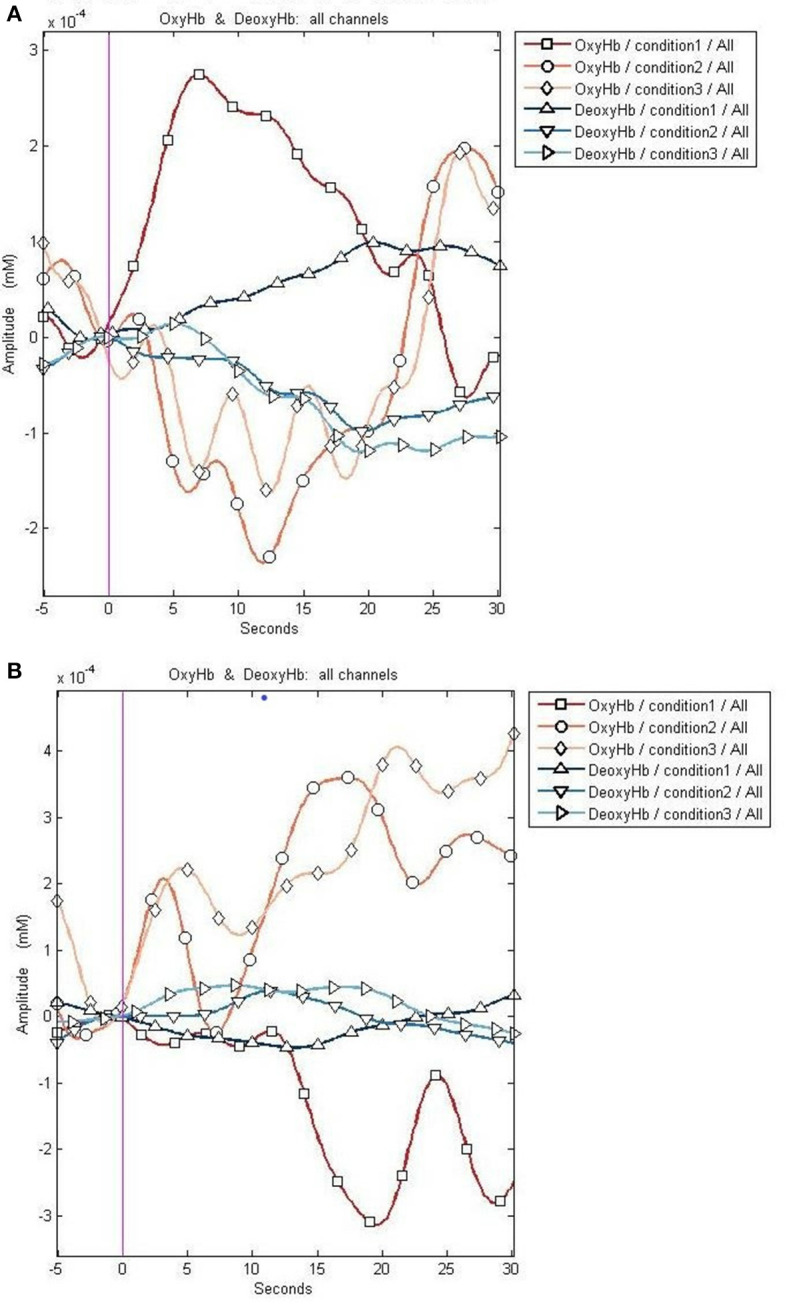
Graphical representation of the changes in the oxy- and deoxyhemoglobin before and after embolization. **(A)** The reduction in oxyhemoglobin after a task (represented by diamonds and circles) before embolization. **(B)** The increase in the oxyhemoglobin after a task (represented by diamonds and circles) after embolization. Deoxyhemoglobin changes appear less prominent compared to oxyhemoglobin changes. The x-axis represents the time course of the changes in hemoglobin concentration, and the y-axis represents the amplitude of the changes in oxy- and deoxyhemoglobin. Condition 1 signifies changes during resting phase. Conditions 2 and 3 demonstrate the changes after a task.

The changes in oxy- and deoxyhemoglobin are demonstrated in Figure 5.

With regard to the prolonged circulation time (>7 s), our study showed an improvement in oxyhemoglobin (−9.51E−05 ± 7.95E−05) in the prolonged circulation time subgroup as compared to the normal circulation time subgroup (6.10E−05 ± 2.92E−04, *p* = 0.07). The changes in deoxyhemoglobin and regional oxygen saturation did not show significant variations (the *p*-values for deoxyhemoglobin and regional oxygen saturation were 0.22 and 0.11, respectively). All three parameters were not significant for other aggressive imaging features, such as hemorrhage or multiple DAVF.

The neuropsychology scores in the patient group exhibited significant improvement after embolization. The MMSE score following embolization was 30 ± 6 (median ± range), and the ACE III score was 85 ± 30 (median ± range). The scores were not significantly correlated with the improvement in postembolization oxyhemoglobin values.

## Discussion

This study aimed to understand the role of fNIRS in evaluating the hemodynamic changes in patients with DAVF. We found a consistent decrease in oxyhemoglobin levels following the Stroop task in the patient group as compared to the control group. The post-Stroop-task oxyhemoglobin levels showed an increase after embolization. The response was more robust in patients with aggressive DAVF. However, deoxyhemoglobin and regional oxygen saturation did not exhibit significant changes after the task. In addition, we noted an overall reduction in neuropsychology scores in patients with DAVF, moreso in those with aggressive DAVF, which suggests the possibility of subclinical cognitive decline in these patients. These scores, too, improved after embolization. The cognitive decline associated with DAVF is reversible and tends to improve within a few months after treatment.

Studies exploring the usefulness of fNIRS for neurological diseases are scarce. There is evidence in the literature for the application of fNIRS in contexts such as traumatic brain injury, epilepsy, Alzheimer's dementia, Parkinson's disease, and stroke rehabilitation. The principle of neurovascular coupling forms the basis for utilizing fNIRS for neurological disorders. Following neural activity, there may be a brief decline in oxyhemoglobin and increase in deoxyhemoglobin, owing to the rapid increase in the cerebral metabolic rate (Racz et al., 2017). Following the brief decline in oxyhemoglobin, the circulation responds with a rapid increase in oxyhemoglobin because of the disproportionate increase in the blood flow, i.e., to rectify the mismatch. This process mirrors the neurovascular coupling of functional MRI (Buxton, 2009); nonetheless, fNIRS measures this subsequent increase in oxyhemoglobin, which indicates the cortical activity.

One drawback of fNIRS is the inclusion of other contaminated signals, such as extracerebral and systemic signals (including variations in heart rate and respiratory rate), which can lead to inaccurate interpretation of the cerebral hemodynamic assessment (Scholkmann et al., 2014). This drawback can be corrected by using adaptive filtering (using a band-pass or low-pass filter), block averaging, and general linear model for statistics. Other correction methods include spatially resolved spectroscopy and the self-calibrating method. The other main drawback is its inability to measure the whole brain cortex with adequate spatial resolution. Hemorrhage in the brain of patients affected with bled DAVFs can be a limitation in obtaining accurate results from fNIRS. However, in those patients in our series who had experienced bleeding, the hemorrhage had occurred far away from the region of fNIRS acquisition (i.e., the prefrontal cortex) and thus could not have impacted the measurements.

DAVF is an unusual neurovascular condition that may present with hemorrhagic or non-hemorrhagic complications that are related to the grade of the fistula. Although hemorrhagic complications are linked to structural abnormalities, such as venous ectasia or dilated venous sacs, the non-hemorrhagic events, such as cognitive decline, are often attributed to the central pathogenesis of venous hypertension. The cognitive decline in patients with DAVF can sometimes show rapid progression, but it can be completely reversed after successful treatment (Wilson et al., 2010; Cheng et al., 2018; Colorado et al., 2018). This reversibility underscores the importance of early detection and timely treatment in reducing patient morbidity. MRI and DSA can neither accurately depict venous hypertension nor adequately assess treatment response. Studies using arterial spin labeling (ASL) and single photon emission computed tomography (SPECT) have previously demonstrated parenchymal hyperperfusion at the fistulous site and hypoperfusion away from it (Noguchi et al., 2010). This distant hypoperfusion has been attributed to retrograde venous drainage (venous congestion) hampering the arterial inflow, (cerebral blood flow—CBF) leading to reduced perfusion (rCBF—relative cerebral blood flow). This reduced perfusion improved after treating the DAVF.

The present study is in line with previous studies that observed significantly reduced oxyhemoglobin (comparable to CBF) in patients with aggressive DAVF, with improvement after endovascular treatment. However, deoxyhemoglobin and regional oxygen saturation did not show significant changes in the patients. One hypothesis is that the venous hypertension associated with DAVF could have increased the resistance of the veins. This resistance could in turn have led to varying degrees of dilation and stenosis in the veins, thus causing variation in the total deoxyhemoglobin content (Irani et al., 2007).

This reasoning could also explain the lack of significance of deoxyhemoglobin observed in this study. The calculation of regional oxygen saturation involves the subtraction of deoxyhemoglobin from oxyhemoglobin (Scholkmann et al., 2014). Deoxyhemoglobin is a negative integer; thus, regional oxygen saturation tends to be a positive value and, hence, might be nonsignificant. A study using ^123^I-iodoamphetamine SPECT has also reported reduced CBF because of the venous congestion in aggressive DAVF (Kanemaru et al., 2015). Furthermore, a case report demonstrated the use of fNIRS in patients with DAVF; an increase in deoxyhemoglobin was observed in the patients, which was reversed after treatment (Shidoh et al., 2014). However, in our study involving a large number of patients, the changes in deoxyhemoglobin and regional oxygen saturation were insignificant.

Previous studies have shown the importance of oxyhemoglobin in other neurological and psychiatric disorders. A study by Serap et al. (2009) revealed oxyhemoglobin reductions in patients with attention deficit hyperactivity disorder, especially in the ventral and dorsolateral prefrontal cortices. In a study on Parkinson's disease by Morishita et al. (2016), the authors compared preoperative and postoperative improvement in motor activity with prefrontal cortex fNIRS scores and concluded that the oxyhemoglobin scores were comparable to the motor scores. Similarly, a study of Alzheimer's disease by Kato et al. indicated the importance of region-specific variations in oxyhemoglobin levels in patients with this disease during a single-word presentation task (Bonilauri et al., 2020). A study on children with prenatal alcohol exposure showed high levels of deoxyhemoglobin using fNIRS and observed that such children tend to have more developmental and behavioral problems than those with normal levels of deoxyhemoglobin (Kable et al., 2020). This study underscored the importance of fNIRS in children. Most of the studies to date have demonstrated the utility of oxyhemoglobin values derived from fNIRS in monitoring tissue oxygenation in various neurological disorders.

The changes in neuropsychology scores observed in our study were comparable to those observed in previous studies on DAVF (Wilson et al., 2010). In addition, this research found a moderately positive correlation between the changes in oxyhemoglobin and MMSE scores before embolization. This finding implies that there may be a significant reduction in oxyhemoglobin values as cognition worsens. However, this finding needs to be validated in a larger population for it to be generalized.

Studies of DAVF using fNIRS are scarce, except for one case report that assessed DAVF hemodynamics using fNIRS. This study, too, did report reduced oxyhemoglobin in the patients, who showed mild improvement after partial embolization and significant improvement after complete embolization (Shidoh et al., 2014).

Our study has established the feasibility of using fNIRS for the bedside evaluation of cerebral hemodynamics in patients with intracranial DAVF. Portability, non-invasiveness, lack of radiation, repeatability, patient comfort, and cost–effectiveness are some of the advantages of this technique. The observations were consistent and reliable and, hence, can be used as a marker to assess the resolution of hemodynamic impairment following DAVF treatment. The encouraging results from our study demonstrate the translation potential of this brain–computer interface tool from research to clinical application.

The strengths of this study are the inclusion of a modest number of patients with a relatively rare disease, its case–control design, the detailed neuropsychological evaluation, and the follow-up assessment of the treated patients, revealing its clinical potential. The limitations include selection bias; dependence on other hemodynamic parameters, which could influence neurovascular coupling; and the fact that the normal and abnormal values of the hemoglobin parameters are yet to be completely assessed. Our study is a relatively early one; with additional studies on this topic, it may be possible to identify cutoff values that can precisely define normal and abnormal values for the hemoglobin parameters. Consistency in the signal response suggests that these hemodynamic parameters may not considerably influence neurovascular coupling. Further studies are required in this area, especially those using the newly developed wearable and wireless fNIRS tool and advanced post-processing software. A larger study involving DAVF patients using fNIRS may help to enhance the significance of the results and make them more generalizable.

## Conclusion

DAVF is a rare disease with altered cerebral hemodynamics, and cognitive evaluation in DAVF remains understated. The utilization of fNIRS in DAVF as a bedside treatment response tool is feasible to a great extent, particularly to assess postintervention venous hypertension. The change in oxyhemoglobin was found to be a significant parameter in differentiating these patients from controls. In addition, this change also helped in assessing treatment response because it normalized after embolization, with the resolution of venous congestion. A moderate correlation was noted between the pre-embolization neuropsychology scores and changes in oxyhemoglobin; nonetheless, a larger study is needed to validate this finding.

## Data availability statement

The datasets presented in this article will be shared upon reasonable request. Requests to access the datasets should be directed to drsanthoshkannath@gmail.com.

## Ethics statement

The studies involving human participants were reviewed and approved by Institute Ethics Committee, SCTIMST. The patients/participants provided their written informed consent to participate in this study.

## Author contributions

SS: study design, acquisition of data, data analysis, data interpretation, statistical analysis, and manuscript drafting and editing. SKK: conceptualization and design, data acquisition and analysis, manuscript preparation, and revision. KMA: data analysis and data interpretation. RM: study design, data analysis, and manuscript revision. CK: conceptualization and manuscript preparation and revision. All authors contributed to the article and approved the submitted version.

## Funding

This research received a grant from the technology development fund of SCTIMST.

## Conflict of interest

The authors declare that the research was conducted in the absence of any commercial or financial relationships that could be construed as a potential conflict of interest.

## Publisher's note

All claims expressed in this article are solely those of the authors and do not necessarily represent those of their affiliated organizations, or those of the publisher, the editors and the reviewers. Any product that may be evaluated in this article, or claim that may be made by its manufacturer, is not guaranteed or endorsed by the publisher.
